# Supplementary material for: Using Non-Pharmaceutical Interventions and High Isolation of Asymptomatic Carriers to Contain the Spread of SARS-CoV-2 in Nursing Homes
Source: Life (Basel). 2022 Jan 26;12(2):180. doi: 10.3390/life12020180 (PMC8877375; doi:10.3390/life12020180)
Supplement: Supplementary file 1 [file life-12-00180-s001.zip › life-1495286-SI.pdf]

Using Non-Pharmaceutical Interventions and High Isolation of Asymptomatic Carriers to Contain the Spread of SARS-CoV-2 in Nursing Homes

Alec J. Schmidt, Yury García, Diego Pinheiro, Thomas A. Reichert, Miriam Nuño.

January 21, 2022

Methods

The objective of this study was to investigate a range of nonpharmaceutical interventions (NPIs) and their consequences in mitigating the impact of COVID-19 outbreaks with increasing severity in a nursing home. This supporting information is organized into four sections: (i) description of the nonpharmaceutical interventions, (ii) description of the mathematical model and simulations, (iii) a global sensitivity analysis, and (iv) results.

Description of Non-Pharmaceutical Interventions

In this study, we adapted a transmission model from our previous work on influenza to investigate the role of common non-pharmaceutical interventions (NPIs) on preventing or mitigating the spread of the novel coronavirus disease (COVID-19). As in Nuño et al. [1], we simulated multiple NPI scenarios within a model nursing home with varying levels of rigor across a range of possible basic reproductive numbers. The NPI scenarios were updated to reflect new recommendations by the Center for Disease Control and Prevention (CDC) [2] and the World Health Organization (WHO [3]). The role of the silent infections in the spread of the virus and the long periods in which an individual can be infectious are two critical features in the transmission of the SARS-CoV-2 virus. Thus, to adapt the model to COVID-19, we added incremental pre-symptomatic isolation rates as variables separate from the NPI scenarios. As the COVID-19 pandemic has progressed during these past few months, guidelines and recommendations have evolved to protect residents and staff in nursing homes and other long-term care facilities (LTCFs). These measures are integrated in four different plans noted as Baseline, Categories 1-2, Categories 3-4, and Category 5. The baseline scenario reflects a case-scenario in the absence of NPIs. Categories increase in rigor from Categories 1-2 up to Category 5 by introducing new NPI methods or increasing the protectiveness of a previously-incorporated NPI. The different control measures analyzed in this study as well as considerations for each plan of action are described below.

1. **Identify and exclude potentially infected residents, staff, or visitors:** If a resident or staff has any symptoms, they are completely isolated. The entrance

of visitors who manifest any sign of infection is also prohibited. We assume that residents who are successfully isolated are at no risk of subsequent transmission within the facility or to contacts outside the nursing home. On the other hand, symptomatic staff are not allowed in the nursing home, but their complete isolation off-site is not controllable and therefore they still risk transmission in the wider community. This measure is considered in all Categories. The reduction in the transmission rate, due to the exclusion of the symptomatic individuals, is captured by the parameter π_i.

2. **Personal protective equipment:** The implementation of the use of protective equipment such as masks, gowns, or special clothing is required for staff and visitors, but not for residents of the home. Previous studies [4–7] showed that the use of protective implements reduced the possibility of exposure to contact with an infected person. For this study, we only considered the use of face mask. The effectiveness of a face mask in reducing transmission is related to the material and type, as well as time and proper use. We set the transmission rate reduction for mask use at 14.6 % (Use of protective gear is captured by ρ_i).

3. **Staff and visitors entering the facility are monitored for elevated temperature:** The effectiveness is defined by the parameter p_i, where i is the index for exposed (E), asymptomatic (A), and infected individuals (I). We assume two scenarios. One in which 50% of exposed and 10% of symptomatic individuals escape the controls (Category 3-4). A second one where only 14% of the exposed and 10% of symptomatic cases are undetected during the screening (Category 5). Baseline scenario and Category 1-2 do not include implementation of temperature checks. Infectious, asymptomatic people will rarely be caught in this step.

4. **Changes in staff working schedule:** For the baseline scenario and Category 1-2, we assume a base staff schedule of 8 h-day for 5 days a week. In Category 3-4, the working schedule is switched from 8 to 12 hours per day for 4 days a week, leaving a 3 day period of without contact with the facility. Finally, in Category 5, this is extended to a 4-days-on/4-days-off cycle.

5. **Restrict visits:** The baseline scenario assumes a 2-hour visit period (per week), and visitors can enter regardless of infectious status. For Category 1-2, the same visit period is considered, but visitors with symptoms are prohibited from entering the facility, and the use of PPE is mandatory. Category 3-4 is slightly more restrictive, visiting periods are only one hour and temperature controls are imposed. Finally, visits are completely restricted in Category 5.

6. **Isolation of asymptomatic individuals:** Asymptomatic people play an important role in the spread of the new virus [8–10]. Although the degree of infectiousness is not yet clear [11–15], recent studies show that those responsible for some propagation events were pre-symptomatic patients, so their early isolation is relevant to contain the pandemic [8–10,16]. When we talk about asymptomatic people isolation, we refer to both cases, they who remain asymptomatic throughout the infectious period and certain people who are pre-symptomatic, who are still in a latency period. For the purpose of this study, we consider that there is no difference in transmission rates between symptomatic and asymptomatic patients [13]. All categories are simulated considering that asymptomatic people are successfully isolated in 0%, 50%, 60%, 70%,

80%, and 90%. η represents the proportion of people who do not isolate themselves and continue to contribute to new infections (1-% of isolated people). Isolation might be controlled inside the facility, but there is not control outside; that is why η is not included in the transmission rate outside the centers (Eq. [2]).

Since SARS-CoV-2 is a new virus, it is not yet known with certainty what is the proportion of people who remain asymptomatic. The percentages can range from 32.5% to 85% according to some studies reported and summarized in [17, 18]. For this study, we set this value equal to 33.3%. So, the proportion of people exposed that progress to infection is m=0.667.

The use of face masks, eye protection, and physical distancing, as well as the isolation of symptomatic residents and staff, are included in all Categories (1-5). Control measures increase with the introduction of different working hours for staff and a restrictive schedule for visitors, temperature control, and a different percentage of early isolation of asymptomatic cases. Interventions and parameters considered in each case are summarized in Table 6.

Epidemic Model: Nonpharmaceutical Interventions

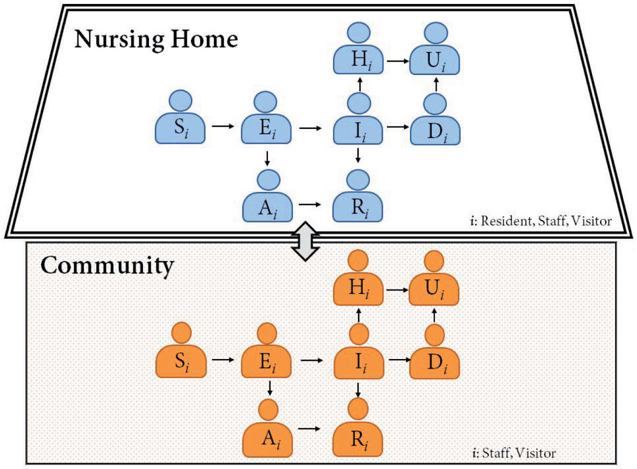

Figure 1: Graphic representation of the interaction between residents and the community in general

To describe the dynamic of the SARS-Cov-2 inside a nursing home, we use an extension of our previous stochastic Susceptible-Exposed-Infected-Recovered (SEIR) model [1]. The care home is part of a wider community, so there are four main actors involved in the dynamic: community members, visitors, staff, and residents. Staff and visitors spend time both inside and outside the facility and can come into contact with residents, other staff and visitors, and community members. Each actor begins susceptible to contracting the virus (S). Once a person is infected, they enter an asymptomatic or pre-symptomatic state (E), where they can become infectious without symptoms [8, 9, 19]. Then, the individual can move to a group of symptomatic individuals (I) or the group of asymptomatic infectious individuals (A). Finally, when the symptoms disappear, and the actor is no longer

infectious, they move to the recovered state. The time in which a person remains immune is still unknown, but for analysis of a single epidemic, we assumed that once an individual recovers, he/she is removed from the system. Each epidemic state is indexed as R for residents; SF and VF for staff (S) and visitors (V) inside the facility (F); SC and VC for staff and visitors in the community and C for people outside the nursing home with no direct contact with residents. Thus, E_{SF} corresponds to an exposed staff inside the facility, A_C is asymptomatic in the community, and so on. The same index scheme is used for parameters. The basic structure of the model remains as previously published [1], the details of which are given in the supplementary material of the previous work. This new study has the additional purpose of studying the required hospital capacity for nursing home residents, we added two new compartments to the respective epidemic models: H_R for people requiring hospitalization and U_R for hospitalized people who move to the intensive care unit. The meaning and values of these parameters are described in the main text.

The infection force inside (Eq. 1) and outside (Eq. 2) the facility described in [1] is modified to include the effect of asymptomatic people on the latency period (E) and the parameters which affect the transmission rate by the interventions described above.

$$\lambda_{in} = \frac{\sum\limits_{i=R,SF,VF} \beta_i \rho_i (\pi_i I_i(t) + \eta(A_i(t) + E_i(i)))}{N_{in}} \tag{1}$$

$$\lambda_{out} = \frac{\sum\limits_{i=C,SC,VC} \beta_i \rho_i (\pi_i I_i(t) + A_i(t) + E_i(t))}{N_{out}} \tag{2}$$

N_{in} corresponds to the nursing home population (residents, staff, and visitors). N_{out} are people outside the facility (staff, visitors, and community). We modeled a facility with a daily average of 200 residents, 83 staff, 40 visitors, in a community with 50,000 individuals.

Stochastic Poisson Simulation of Disease Progression

We solve the stochastic model via a Poisson simulation [20] and consider scenarios that varied the basic reproduction number R_0 in a range from 2 to 4, according to the values reported in the literature [21]. An incremental change of 0.06 in β produced an incremental change of of 0.2 in R_0 (Tab. 1). We ran 100 simulations of each combination of R_0, intervention scenario, and asymptomatic isolation in a epidemic period of $T = 200$ days. Calculation of R_0 is described in [1].

Table 1: R_0 values and transmission rate (β) implemented in the simulations

R_0	2.0	2.2	2.4	2.6	2.8	3.0	3.2	3.4	3.6	3.8	4.0
β	0.61	0.67	0.73	0.79	0.85	0.91	0.97	1.03	1.09	1.15	1.21

Global Sensitivity Analysis

The *ODEsensitivity* R library (v1.1.2; Weber, Theers, and Surmann, 2019) was used for sensitivity analysis. We calculated the first-order Sobol' index for R_0 and the percentage of asymptomatic isolation to study the contribution of each to the attack rate, mortality rate, hospitalizations, and ICU admission for residents. The Sobol' method allows more precision to detect the most influential parameters in the model [22]. The analysis reveal that model outputs were generally more sensitive to the proportion of asymptomatic patients who are isolated than to the value of R_0 (Fig. 2), which is in correspondence with the results described in the main text. If a significant proportion of asymptomatic patients are not isolated, the outbreak will always be out of control, regardless of the value of R_0 (Figures. 4, 3).

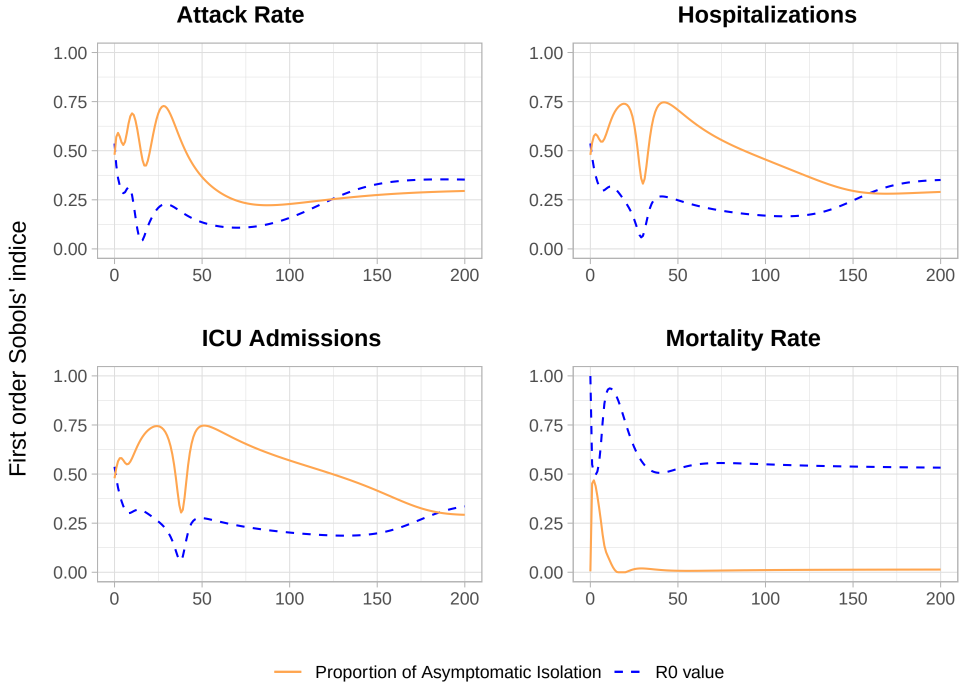

Figure 2: The temporal variation of the sensitivity of the infected population size (attack rate), hospitalization, ICU and fatality cases to R_0 values and proportion of asymptomatic isolation

Results

Figure 4 and 3 correspond to the result of simulating a combination of R_0 values with the different categories of intervention with 0% and 50% of asymptomatic isolation rate. The results indicate that even if strict NPIs are implemented, control of the spread of the virus is not achieved without extremely high rates of excluding asymptomatic individuals. When 50% of the silent transmissions are isolated, NPIs have a small effect on virus containment at low values of R_0 which rapidly disappeared as R_0 increased.

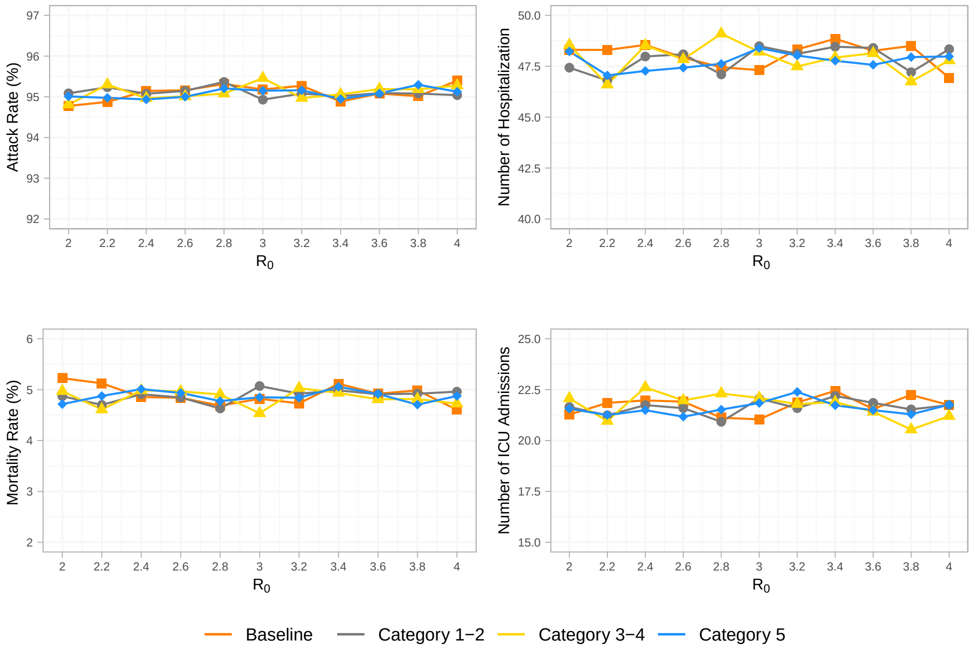

Figure 3: Attack rate, mortality rate, hospital and ICU admissions at 0% isolation

Table 2: Means and 95% CIs for the four main outcomes at 0% isolation

	Baseline					Category 1-2				
R_0	Epidemic Size	Hospitalization	ICU	Death	Staff	Epidemic Size	Hospitalization	ICU	Death	Staff
2.0	(188, 190, 196)	(45, 48, 61)	(18, 21, 30)	(8, 10, 17)	(25, 28, 36)	(188, 190, 196)	(44, 47, 60)	(19, 22, 30)	(8, 10, 15)	(26, 29, 36)
2.2	(187, 190, 196)	(44, 48, 64)	(19, 22, 31)	(8, 10, 18)	(25, 29, 36)	(189, 190, 196)	(43, 47, 59)	(18, 21, 32)	(7, 9, 16)	(25, 28, 36)
2.4	(188, 190, 196)	(44, 49, 62)	(19, 22, 30)	(8, 9, 16)	(26, 28, 36)	(188, 190, 196)	(44, 48, 60)	(19, 22, 31)	(8, 10, 15)	(25, 28, 36)
2.6	(189, 190, 195)	(44, 48, 58)	(19, 22, 31)	(8, 10, 14)	(25, 29, 39)	(188, 190, 195)	(44, 49, 59)	(19, 22, 30)	(8, 10, 16)	(26, 29, 36)
2.8	(189, 191, 195)	(43, 48, 59)	(18, 21, 30)	(7, 9, 16)	(25, 28, 38)	(189, 191, 196)	(42, 47, 60)	(18, 21, 29)	(8, 9, 15)	(24, 28, 37)
3.0	(189, 190, 196)	(44, 47, 57)	(18, 21, 33)	(8, 10, 16)	(26, 28, 36)	(188, 190, 196)	(45, 48, 59)	(19, 22, 32)	(8, 10, 17)	(26, 29, 37)
3.2	(189, 191, 196)	(45, 48, 63)	(19, 22, 31)	(8, 9, 15)	(26, 29, 37)	(189, 190, 196)	(44, 48, 60)	(19, 22, 29)	(8, 10, 16)	(27, 29, 37)
3.4	(187, 190, 196)	(45, 49, 61)	(20, 22, 34)	(8, 10, 17)	(26, 29, 36)	(188, 190, 196)	(44, 48, 60)	(19, 22, 33)	(8, 10, 15)	(26, 29, 36)
3.6	(188, 190, 195)	(45, 48, 62)	(19, 22, 30)	(7, 10, 17)	(25, 28, 37)	(188, 190, 196)	(44, 48, 61)	(19, 22, 34)	(7, 10, 17)	(25, 28, 37)
3.8	(188, 190, 195)	(45, 49, 60)	(19, 22, 30)	(8, 10, 16)	(25, 28, 37)	(189, 190, 196)	(44, 47, 58)	(19, 22, 31)	(8, 10, 16)	(26, 28, 35)
4.0	(188, 190, 195)	(45, 48, 58)	(19, 22, 31)	(8, 10, 16)	(25, 28, 38)	(188, 191, 195)	(43, 48, 62)	(19, 21, 30)	(7, 9, 15)	(39, 43, 52)

	Category3-4					Category 5				
R_0	Epidemic Size	Hospitalization	ICU	Death	Staff	Epidemic Size	Hospitalizatio	ICU	Death	Staff
2.0	(188, 190, 196)	(44, 49, 59)	(20, 22, 31)	(8, 10, 16)	(40, 43, 52)	(188, 190, 196)	(43, 48, 61)	(19, 22, 32)	(8, 9, 16)	(39, 43, 52)
2.2	(189, 191, 196)	(42, 47, 59)	(18, 21, 29)	(7, 9, 16)	(39, 42, 52)	(188, 190, 196)	(41, 47, 60)	(18, 21, 33)	(8, 10, 15)	(39, 43, 52)
2.4	(188, 190, 196)	(45, 49, 61)	(19, 23, 33)	(8, 10, 16)	(39, 42, 51)	(188, 190, 195)	(44, 47, 57)	(19, 21, 28)	(8, 10, 16)	(39, 43, 54)
2.6	(189, 190, 195)	(43, 48, 63)	(19, 22, 32)	(8, 10, 17)	(38, 42, 51)	(188, 190, 196)	(43, 47, 60)	(18, 21, 32)	(8, 10, 18)	(39, 42, 51)
2.8	(188, 190, 196)	(45, 49, 63)	(19, 22, 31)	(8, 10, 15)	(40, 43, 53)	(188, 190, 196)	(44, 48, 59)	(19, 22, 32)	(7, 10, 15)	(40, 43, 52)
3.0	(189, 191, 197)	(45, 48, 59)	(19, 22, 29)	(7, 9, 14)	(38, 42, 53)	(188, 190, 196)	(43, 48, 61)	(19, 22, 31)	(7, 10, 16)	(41, 43, 53)
3.2	(188, 190, 196)	(43, 47, 58)	(19, 22, 32)	(8, 10, 17)	(39, 42, 51)	(188, 190, 196)	(44, 48, 59)	(19, 22, 30)	(7, 10, 16)	(40, 42, 51)
3.4	(188, 190, 195)	(45, 48, 60)	(19, 22, 33)	(8, 10, 15)	(40, 43, 52)	(188, 190, 196)	(44, 48, 60)	(19, 22, 31)	(8, 10, 17)	(39, 42, 50)
3.6	(188, 190, 196)	(44, 48, 61)	(18, 21, 30)	(7, 10, 16)	(40, 43, 50)	(188, 190, 195)	(43, 48, 60)	(18, 21, 30)	(7, 10, 15)	(40, 43, 50)
3.8	(188, 190, 196)	(43, 47, 59)	(17, 21, 27)	(8, 10, 15)	(40, 42, 52)	(189, 191, 196)	(44, 48, 59)	(18, 21, 29)	(8, 9, 15)	(39, 42, 52)
4.0	(188, 191, 195)	(43, 48, 62)	(19, 21, 30)	(7, 9, 15)	(39, 43, 52)	(188, 190, 196)	(44, 48, 59)	(19, 22, 30)	(8, 10, 16)	(38, 42, 51)

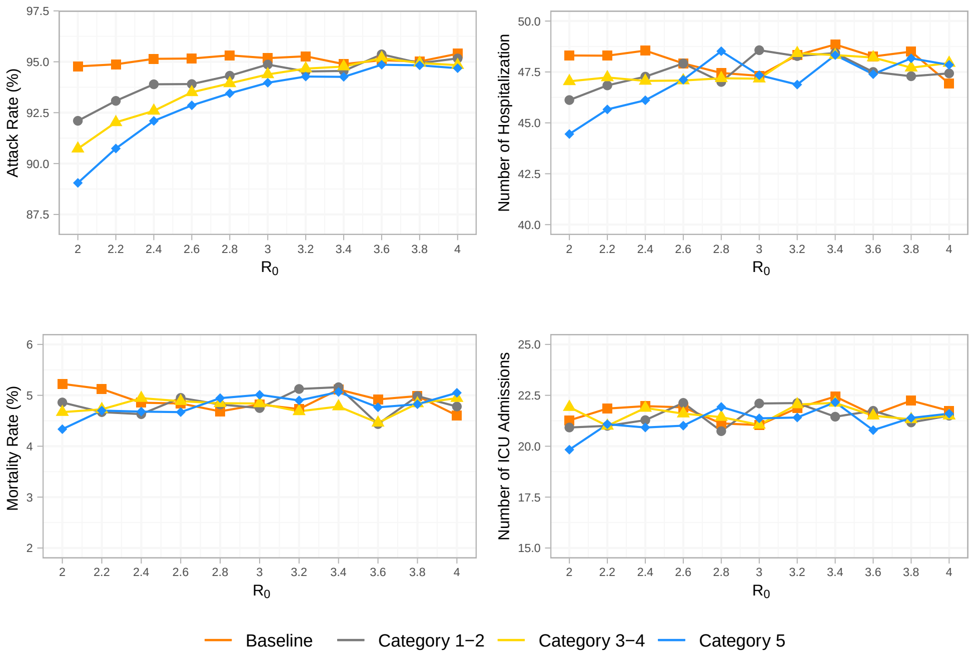

Figure 4: Attack rate, mortality rate, hospital and ICU admissions at 50% isolation

Table 3: Means and 95% CIs for the four main outcomes at 50% isolation

	Baseline					Category 1-2				
R_0	Epidemic Size	Hospitalization	ICU	Death	Staff	Epidemic Size	Hospitalization	ICU	Death	Staff
2.0	(188, 189, 196)	(45, 48, 61)	(18, 21, 30)	(8, 10, 17)	(25, 28, 36)	(181, 184, 192)	(43, 46, 57)	(18, 21, 29)	(8, 10, 16)	(25, 29, 38)
2.2	(187, 190, 196)	(44, 48, 64)	(19, 22, 31)	(8, 10, 18)	(25, 29, 36)	(184, 186, 194)	(43, 47, 57)	(18, 21, 30)	(8, 9, 15)	(25, 28, 38)
2.4	(188, 190, 196)	(44, 49, 62)	(19, 22, 30)	(8, 10, 16)	(26, 28, 36)	(186, 188, 193)	(43, 47, 60)	(19, 21, 29)	(8, 9, 16)	(25, 28, 37)
2.6	(189, 190, 195)	(44, 48, 58)	(19, 22, 31)	(8, 10, 14)	(25, 29, 39)	(186, 188, 194)	(44, 48, 58)	(19, 22, 30)	(8, 10, 15)	(25, 29, 38)
2.8	(189, 191, 195)	(43, 47, 59)	(18, 21, 30)	(7, 9, 16)	(25, 28, 38)	(187, 189, 195)	(43, 47, 59)	(17, 21, 29)	(7, 10, 19)	(26, 28, 37)
3.0	(189, 190, 196)	(44, 47, 57)	(18, 21, 33)	(8, 10, 16)	(26, 28, 36)	(188, 190, 195)	(45, 49, 59)	(19, 22, 32)	(7, 10, 17)	(25, 28, 36)
3.2	(189, 191, 196)	(45, 48, 63)	(19, 22, 31)	(8, 9, 15)	(26, 29, 37)	(187, 189, 195)	(43, 48, 61)	(19, 22, 31)	(8, 10, 17)	(25, 28, 36)
3.4	(187, 190, 196)	(45, 49, 61)	(20, 22, 34)	(8, 10, 17)	(26, 29, 36)	(187, 189, 194)	(45, 48, 60)	(18, 21, 30)	(8, 10, 16)	(26, 29, 37)
3.6	(188, 190, 195)	(45, 48, 62)	(19,22, 30)	(7,10, 17)	(25, 28, 37)	(189, 191, 196)	(44, 48, 60)	(18, 22,30)	(7,9, 15)	(25, 29, 36)
3.8	(188, 190, 195)	(45, 49, 60)	(19, 22, 30)	(8, 10, 16)	(25, 28, 37)	(188, 189.87, 195)	(42, 47, 59)	(19, 21, 30)	(8, 10, 17)	(25, 28, 36)
4.0	(189, 191, 195)	(42, 47, 57)	(19, 22, 29)	(7, 9, 15)	(26, 29, 39)	(188, 190, 196)	(43, 47, 60)	(19, 21, 30)	(7,10, 15)	(24, 28, 36)
	Category3-4					Category 5				
R_0	Epidemic Size	Hospitalization	ICU	Death	Staff	Epidemic Size	Hospitalization	ICU	Death	Staff
2.0	(178, 181, 191)	(43, 47, 60)	(19, 22, 31)	(7, 9, 16)	(39, 42, 51)	(175, 178, 188)	(40, 44, 57)	(17, 20, 29)	(7, 9, 15)	(40, 43, 51)
2.2	(181, 184, 192)	(43, 47, 61)	(18, 21, 28)	(7, 9, 16)	(40, 43, 52)	(179, 181, 191)	(41, 46, 59)	(18, 21, 29)	(7, 9, 17)	(40, 43, 52)
2.4	(183, 185, 191)	(43, 47, 60)	(18, 22, 32)	(8, 10, 16)	(39, 43, 52)	(182, 184, 192)	(42, 46, 57)	(18, 21, 28)	(7, 9, 16)	(39, 42, 51)
2.6	(185, 187, 193)	(43, 47, 58)	(18, 22, 30)	(7, 10, 16)	(39, 42, 52)	(184, 186, 192)	(43, 47, 59)	(18, 21, 29)	(8, 9, 14)	(39, 42, 50)
2.8	(185, 188, 194)	(43, 47, 57)	(18, 21, 30)	(8, 10, 15)	(39, 43, 51)	(184, 187 194)	(45, 49, 58)	(20, 22, 30)	(8, 10, 16)	(39, 43, 50)
3.0	(186, 189, 195)	(43, 47, 58)	(18, 21, 30)	(8, 10, 17)	(39, 43, 52)	(185, 188, 195)	(43, 47, 60)	(18, 21, 30)	(8, 10, 17)	(40, 43, 52)
3.2	(188, 189, 195)	(44, 48, 61)	(19, 22, 31)	(7, 9, 16)	(39, 42, 51)	(186, 189, 196)	(43, 47, 59)	(19, 21, 31)	(7, 10, 16)	(40, 43, 51)
3.4	(187, 190, 195)	(44, 48, 60)	(19, 22, 32)	(7, 10, 16)	(39, 43, 53)	(186, 189, 194)	(44, 48, 62)	(19, 22, 31)	(8, 10, 17)	(40, 43, 53)
3.6	(189, 190, 196)	(45, 48, 59)	(19, 22, 32)	(7, 9, 15)	(40, 42, 50)	(188, 190, 195)	(43, 47, 58)	(18, 21, 29)	(8, 10, 16)	(39, 42, 50)
3.8	(188, 190, 196)	(44, 47, 63)	(18, 21, 31)	(8, 10, 18)	(39, 43, 51)	(188, 190, 197)	(44, 48, 61)	(19, 21, 29)	(8, 10, 17)	(39, 42, 49)
4.0	(188, 190, 195)	(45, 48, 58)	(19, 22, 30)	(8, 10, 17)	(40, 43, 50)	(187, 189, 195)	(43, 48, 60)	(19, 22, 32)	(8, 10, 16)	(38, 42, 52)

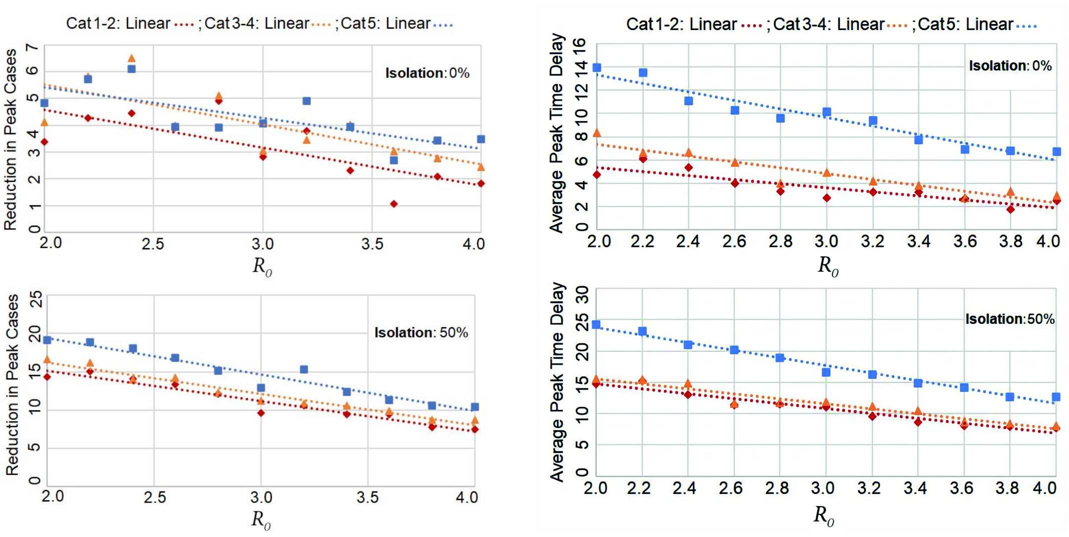

Figure 5: **Left panel:** Difference in average peak outbreak size from baseline scenario at 0% and 50% isolation rate. **Right Panel:** Peak Time delay for NPI scenarios compared to baseline at 0% and 50% isolation rate.

Table 4: Means and 95% CIs for the four main outcomes at 90% isolation

	Baseline					Category 1-2				
R_0	Epidemic Size	Hospitalization	ICU	Death	Staff	Epidemic Size	Hospitalization	ICU	Death	Staff
2.0	(188, 190, 196)	(45, 48, 61)	(18, 21, 30)	(8, 10, 17)	(25, 28, 36)	(48, 57, 81)	(11, 15, 24)	(5, 7, 13)	(2, 3, 6)	(25, 28, 36)
2.2	(187, 190, 196)	(44, 48, 64)	(19, 22, 31)	(8, 10,18)	(25, 29, 36)	(54, 64, 86)	(13, 17 25)	(6, 8, 15)	(2, 4, 7)	(26, 28, 37)
2.4	(188, 190, 196)	(44, 49, 62)	(19, 22, 30)	(8, 10, 16)	(26, 28, 36)	(61, 71, 96)	(14, 18, 28)	(6, 8, 15)	(2, 3, 8)	(25, 28, 36)
2.6	(189, 190, 195)	(44, 48, 58)	(19, 22, 31)	(8, 10, 14)	(25, 29, 39)	(69, 77, 102)	(16, 20, 32)	(6, 9, 16)	(2, 4, 9)	(26, 28, 37)
2.8	(189, 191, 195)	(43, 47, 59)	(18, 21, 30)	(7, 9, 16)	(25, 28, 38)	(77, 85, 109)	(18, 22, 33)	(7, 10, 18)	(3, 4, 10)	(25, 28, 38)
3.0	(189, 190, 196)	(44, 47, 57)	(18, 21, 33)	(8, 10, 16)	(26, 28, 36)	(82, 93, 122)	(20, 24, 34)	(8, 11, 18)	(4, 5, 10)	(25, 28, 35)
3.2	(189, 191, 196)	(45, 48, 63)	(19, 22, 31)	(8, 9, 15)	(26, 29, 37)	(92, 101, 129)	(23, 26, 39)	(9, 12, 20)	(4, 5, 10)	(25, 28, 36)
3.4	(187, 190, 196)	(45, 49, 61)	(20, 22, 34)	(8, 10, 17)	(26, 29, 36)	(102, 111, 140)	(24, 28, 38)	(10, 12, 19)	(4, 6, 11)	(26, 29, 39)
3.6	(188, 190, 195)	(45, 48, 62)	(19, 22, 30)	(7, 10, 17)	(25, 28, 37)	(105, 113, 137)	(25, 29, 41)	(10, 13, 20)	(4, 6, 11)	(26, 29, 38)
3.8	(188, 190, 195)	(45, 49, 60)	(19, 22, 30)	(8, 10, 16)	(25, 28, 37)	(112, 120, 141)	(26, 30, 41)	(11, 14, 21)	(4, 6, 12)	(26, 29, 37)
4.0	(189, 191, 195)	(42, 47, 57)	(19, 22, 29)	(7, 9, 15)	(26, 29, 39)	(117, 125, 150)	(26, 31, 42)	(12, 14, 21)	(4, 6, 12)	(26, 29, 35)

	Category3-4					Category 5				
R_0	Epidemic Size	Hospitalization	ICU	Death	Staff	Epidemic Size	Hosp	ICU	Death	Staff
2.0	(30, 38, 62)	(7, 10, 17)	(3, 5, 10)	(1, 2, 5)	(40, 43, 54)	(22, 28, 46)	(5, 7, 13)	(2, 3, 7)	(1, 2, 4)	(39, 42, 50)
2.2	(38, 47, 71)	(9, 12, 21)	(4, 6, 12)	(1, 3, 6)	(39, 42, 49)	(24, 34, 58)	(6, 9, 18)	(2, 4, 9)	(1, 2, 5)	(39, 42, 52)
2.4	(44, 53, 84)	(10, 13, 21)	(4, 6, 11)	(1, 3, 6)	(40, 42, 52)	(27, 36, 54)	(6, 9, 17)	(2, 4, 10)	(1, 2, 6)	(39, 42, 51)
2.6	(50, 60, 87)	(12, 15, 24)	(5, 7, 14)	(2, 3, 8)	(40, 43, 50)	(32, 41, 71)	(8, 11, 20)	(3, 5, 10)	(1, 2, 6)	(38, 42, 51)
2.8	(57, 67, 96)	(14, 17, 27)	(5, 8, 15)	(2, 4, 9)	(40, 43, 51)	(39, 51, 80)	(8, 13, 25)	(3, 6, 14)	(1, 3, 7)	(39, 43, 51)
3.0	(58, 68, 98)	(14, 18, 28)	(6, 8, 14)	(2, 3, 8)	(40, 42, 52)	(48, 59, 97)	(11, 15, 26)	(5, 7, 15)	(2, 3, 9)	(39, 42, 51)
3.2	(69, 80, 110)	(16, 19, 30)	(7, 9, 14)	(2, 4, 8)	(40, 43, 51)	(49, 60, 97)	(10, 15, 25)	(5, 7, 12)	(2, 3, 7)	(39, 42, 52)
3.4	(73, 83, 112)	(17, 21, 35)	(7, 10, 19)	(3, 5, 10)	(39, 43, 51)	(50, 66, 100)	(13, 17, 28)	(5, 7, 15)	(2, 4, 8)	(39, 43, 55)
3.6	(83, 95, 130)	(20, 24, 35)	(9, 11, 18)	(3, 5, 9)	(40, 43, 51)	(63, 74.2, 111)	(15, 18, 30)	(6, 8, 15)	(2, 4, 8)	(40, 43, 52)
3.8	(86, 102, 142)	(21, 25, 36)	(9, 11, 19)	(3, 5, 11)	(39, 43, 52)	(68, 78, 110)	(16, 20, 35)	(7, 9, 16)	(3, 4, 10)	(40, 42, 51)
4.0	(97, 105, 134)	(24, 27, 40)	(10, 12, 18)	(4, 6, 11)	(40, 43, 52)	(77, 86, 118)	(17, 21, 36)	(7, 10, 18)	(2, 4, 8)	(40, 43, 52)

Table 5: Attack rate for different percentage of asymptomatic isolation

Attack Rate- Category5

R_0	0%	50%	60%	70%	72%	74%	76%	78%	80%	82%	84%	86%	88%	90%
2.0	190	178	166	138	135	100	111	98	83	73	58	47	37	28
2.2	190	181	172	153	145	136	127	113	104	86	70	53	43	34
2.4	190	184	176	158	154	152	135	128	114	96	80	66	51	36
2.6	190	186	180	164	160	152	144	133	123	109	93	71	61	41
2.8	190	187	183	171	166	157	152	144	132	119	103	86	69	51
3.0	190	188	185	173	170	160	159	1501	141	129	115	94	75	59
3.2	190	189	186	177	174	173	161	157	147	134	120	102	81	60
3.4	190	189	186	179	177	170	168	162	153	143	129	111	86	66
3.6	190	190	187	181	179	175	171	167	158	149	137	122	98	74
3.8	191	190	189	183	181	183	174	169	162	152	142	124	104	78
4.0	190	189	189	184	183	184	176	173	165	158	148	131	110	86
**Attack Rate - Category 1-2**														
2.0	190	184	179	162	157	151	142	138	124	113	99	86	71	58
3.0	190	189	188	181	179	178	173	169	162	154	143	129	112	92
4.0	190	190	189	187	186	185	183	180	178	173	166	157	141	124
**Attack Rate - Category 3-4**														
2.0	190	181	173	150	143	134	130	116	105	95	80	63	52	42
3.0	189	189	186	177	175	171	166	159	152	145	127	111	95	71
4.0	190	190	189	186	184	182	181	177	172	165	154	142	127	104

Table 6: Prevention measures and parameters for each scenario

**Baseline**	**Category 1-2**	**Category 3-4**	**Category 5**
Identify and exclude potentially infected residents, staff, or visitors (π_i = Reduction of the transmission rate due to the isolation)			
No isolation	Complete isolation	Complete isolation	Complete isolation
$\pi_R = 1$	$\pi_R = 0$	$\pi_R = 0$	$\pi_R = 0$
$\pi_{SF} = 1$	$\pi_{SF} = 0$	$\pi_{SF} = 0$	$\pi_{SF} = 0$
$\pi_{VF} = 1$	$\pi_{VF} = 0$	$\pi_{VF} = 0$	$\pi_{VF} = 0$
Implementation of the use of protection such as masks, gowns or special clothing for staff and visitors (ρ_i = Reduction of transmission due to these prevention measures, $i \in \{SF, VF, SC, VF, C\}$)			
No required	Required	Required	Required
$\rho_R = 1$	$\rho_R = 1$	$\rho_R = 1$	$\rho_R = 1$
$\rho_i = 1$	$\rho_i = 0.86$	$\rho_i = 0.86$	$\rho_i = 0.86$
Staff and visitors entering a facility is monitored for elevated temperature *(p_I, p_E, p_A = probability of having symptoms, exposed, or asymptomatic escape monitoring efforts)*			
No control	No control	50% of exposed and 10% of infected escape	14% of exposed and 10% of infected escape
$p_E = 1$	$p_E = 1$	$p_E = 0.5$	$p_E = 0.14$
$p_I = 1$	$p_I = 1$	$p_I = 0.1$	$p_I = 0.1$
$p_A = 1$	$p_A = 1$	$p_A = 1$	$p_A = 1$
Staff working schedule ($1/\tau_{SF}, 1/\tau_{SC}$ = Average time spent between locations by staff)			
8-h (5 days)	8-h (5 days)	12-h per day	4-days-on / 4-days-off- site
$\tau_{SF} = 3\ day^{-1}$	$\tau_{SF} = 3\ day^{-1}$	$\tau_{SF} = 2\ day^{-1}$	$\tau_{SF} = 1/4\ day^{-1}$
$\tau_{SC} = 3\ day^{-1}$	$\tau_{SC} = 3\ day^{-1}$	$\tau_{SC} = 2\ day^{-1}$	$\tau_{SC} = 1/4\ day^{-1}$
Visiting periods ($1/\tau_{VF}, 1/\tau_{VC}$ = Average time spent between locations by visitor)			
2h/week	2h/week	1h/week	No visits
$\tau_{VF} = 12\ day^{-1}$	$\tau_{VF} = 12\ day^{-1}$	$\tau_{VF} = 24\ day^{-1}$	$\tau_{VF} = 0\ day^{-1}$
$\tau_{VC} = 0.1428\ day^{-1}$	$\tau_{VC} = 0.1428\ day^{-1}$	$\tau_{VC} = 0.1428\ day^{-1}$	$\tau_{VC} = 0\ day^{-1}$

References

[1] M. Nuño, T. A. Reichert, G. Chowell, and A. B. Gumel, "Protecting residential care facilities from pandemic influenza," *Proceedings of the National Academy of Sciences*, vol. 105, no. 30, pp. 10625–10630, 2008.

[2] C. for Disease Control and Prevention, "Preparing for covid-19 in nursing homes — cdc," June 2020.

[3] W. H. Organization, "Who-2019-ncov-ipc_long_term_care-2020.1-eng.pdf," March 2020.

[4] D. Chu, E. Akl, A. El-Harakeh, A. Bognanni, T. Lotf, M. Loeb, A. Hajizadeh, A. Bak, A. Izcovich, C. A. Cuello-Garcia, *et al.*, "Physical distancing, face masks, and eye protection to prevent person-person covid-19 transmission: A systematic review and meta-analysis," 2020.

[5] T. Li, Y. Liu, M. Li, X. Qian, and S. Y. Dai, "Mask or no mask for covid-19: A public health and market study," *PloS one*, vol. 15, no. 8, p. e0237691, 2020.

[6] M. van der Sande, P. Teunis, and R. Sabel, "Professional and home-made face masks reduce exposure to respiratory infections among the general population," *PloS one*, vol. 3, no. 7, p. e2618, 2008.

[7] C. N. Ngonghala, E. Iboi, S. Eikenberry, M. Scotch, C. R. MacIntyre, M. H. Bonds, and A. B. Gumel, "Mathematical assessment of the impact of non-pharmaceutical interventions on curtailing the 2019 novel coronavirus," *Mathematical Biosciences*, p. 108364, 2020.

[8] M. M. Arons, K. M. Hatfield, S. C. Reddy, A. Kimball, A. James, J. R. Jacobs, J. Taylor, K. Spicer, A. C. Bardossy, L. P. Oakley, *et al.*, "Presymptomatic sars-cov-2 infections and transmission in a skilled nursing facility," *New England journal of medicine*, 2020.

[9] S. M. Moghadas, M. C. Fitzpatrick, P. Sah, A. Pandey, A. Shoukat, B. H. Singer, and A. P. Galvani, "The implications of silent transmission for the control of covid-19 outbreaks," *Proceedings of the National Academy of Sciences*, 2020.

[10] R. Li, S. Pei, B. Chen, Y. Song, T. Zhang, W. Yang, and J. Shaman, "Substantial undocumented infection facilitates the rapid dissemination of novel coronavirus (sars-cov-2)," *Science*, vol. 368, no. 6490, pp. 489–493, 2020.

[11] C. for Disease Control and Prevention, "Management of patients with confirmed 2019-ncov — cdc," June 2020.

[12] A. J. Kucharski, P. Klepac, A. Conlan, S. M. Kissler, M. Tang, H. Fry, J. Gog, J. Edmunds, C. C.-. W. Group, *et al.*, "Effectiveness of isolation, testing, contact tracing and physical distancing on reducing transmission of sars-cov-2 in different settings," *medRxiv*, 2020.

[13] G. Yin and H. Jin, “Comparison of transmissibility of coronavirus between symptomatic and asymptomatic patients: Reanalysis of the ningbo covid-19 data,” *JMIR Public Health and Surveillance*, vol. 6, no. 2, p. e19464, 2020.

[14] L. Zou, F. Ruan, M. Huang, L. Liang, H. Huang, Z. Hong, J. Yu, M. Kang, Y. Song, J. Xia, *et al.*, “Sars-cov-2 viral load in upper respiratory specimens of infected patients,” *New England Journal of Medicine*, vol. 382, no. 12, pp. 1177–1179, 2020.

[15] X. He, E. H. Lau, P. Wu, X. Deng, J. Wang, X. Hao, Y. C. Lau, J. Y. Wong, Y. Guan, X. Tan, *et al.*, “Temporal dynamics in viral shedding and transmissibility of covid-19,” *Nature medicine*, vol. 26, no. 5, pp. 672–675, 2020.

[16] J. Hellewell, S. Abbott, A. Gimma, N. I. Bosse, C. I. Jarvis, T. W. Russell, J. D. Munday, A. J. Kucharski, W. J. Edmunds, F. Sun, *et al.*, “Feasibility of controlling covid-19 outbreaks by isolation of cases and contacts,” *The Lancet Global Health*, 2020.

[17] D. H. M. K. C. V. R. B. R. M. Roy Anderson, Christl Donnelly, “Reproduction number (r) and growth rate (r) of the covid-19 epidemic in the uk.” `https://royalsociety.org/-/media/policy/projects/set-c/set-covid-19-R-estimates.pdf?la=en-GB&hash=FDFFC11968E5D247D8FF641930680BD6`, August 2020. (Accessed on 10/01/2020).

[18] W. J. Wiersinga, A. Rhodes, A. C. Cheng, S. J. Peacock, and H. C. Prescott, “Pathophysiology, transmission, diagnosis, and treatment of coronavirus disease 2019 (covid-19): a review,” *Jama*, 2020.

[19] T. M. McMichael, D. W. Currie, S. Clark, S. Pogosjans, M. Kay, N. G. Schwartz, J. Lewis, A. Baer, V. Kawakami, M. D. Lukoff, *et al.*, “Epidemiology of covid-19 in a long-term care facility in king county, washington,” *New England Journal of Medicine*, vol. 382, no. 21, pp. 2005–2011, 2020.

[20] L. Gustafsson, “Poisson simulation—a method for generating stochastic variations in continuous system simulation,” *Simulation*, vol. 74, no. 5, pp. 264–274, 2000.

[21] Y. Liu, A. A. Gayle, A. Wilder-Smith, and J. Rocklöv, “The reproductive number of covid-19 is higher compared to sars coronavirus,” *Journal of travel medicine*, 2020.

[22] J. Wu, R. Dhingra, M. Gambhir, and J. V. Remais, “Sensitivity analysis of infectious disease models: methods, advances and their application,” *Journal of The Royal Society Interface*, vol. 10, no. 86, p. 20121018, 2013.
